# TLR9 Ligands Induce S100A8 in Macrophages via a STAT3-Dependent Pathway which Requires IL-10 and PGE_2_

**DOI:** 10.1371/journal.pone.0103629

**Published:** 2014-08-06

**Authors:** Kenneth Hsu, Yuen Ming Chung, Yasumi Endoh, Carolyn L. Geczy

**Affiliations:** Inflammation and Infection Research Centre, School of Medical Sciences, University of New South Wales, Sydney, New South Wales, Australia; University of Tokyo, Japan

## Abstract

S100A8 and S100A9 are highly-expressed calcium-binding proteins in neutrophils and monocytes, and in subsets of macrophages in inflammatory lesions. Unmethylated CpG motifs found in bacterial and viral DNA are potent activators of innate immunity via Toll-like receptor 9 (TLR9). S100A8, but not S100A9, mRNA and protein was directly induced by CpG-DNA in murine and human macrophages. Induction in murine macrophages peaked at 16 h. CpG-DNA-induced S100A8 required *de novo* protein synthesis; IL-10 and Prostaglandin E_2_ (PGE_2_) synergistically enhanced expression and promoted earlier gene induction. Inhibitors of endogenous IL-10, PGE_2_, and the E prostanoid (EP) 4 receptor strongly suppressed S100A8 expression, particularly when combined. Thus, S100A8 induction by *E. coli* DNA required both IL-10 and PGE_2_/EP4 signaling. The MAPKs, PI3K and JAK pathways were essential, whereas ERK1/2 appeared to play a direct role. S100A8 induction by CpG-DNA was controlled at the transcriptional level. The promoter region responsible for activation, either directly, or indirectly via IL-10 and PGE_2_, was located within a −178 to −34-bp region and required STAT3 binding. Because of the robust links connecting IL-10 and PGE_2_ with an anti-inflammatory macrophage phenotype, the induction profile of S100A8 strongly indicates a role for this protein in resolution of inflammation.

## Introduction

Responses in the innate immune system in vertebrates rely on germline-encoded pattern recognition receptors (PRRs), including the Toll-like receptor (TLR) family, to sense pathogen-associated molecular patterns (PAMPs) on microbial pathogens. Macrophages are crucial mediators in this process, producing proinflammatory cytokines, chemokines and antimicrobial proteins in response to PRR signaling [1]. Response genes in activated macrophages can be fundamentally divided into primary (0.5–2 h, e.g. TNFα) and secondary response genes (2–8 h, e.g. IL-10) (reviewed in [2]) that may display differences in their chromatin architecture (e.g. presence of CpG islands) and/or their regulation by different categories of transcription factors [3]. Secondary genes induced by LPS generally require *de novo* synthesis and further chromatin remodelling that regulates subsequent waves of late gene expression over a prolonged period, thus determining the fate of activated macrophages. However, mechanisms involved in generation of late-phase responses by secondary response genes are poorly defined.

Bacterial and viral DNAs are PAMPs, distinct from their vertebrate counterparts, containing unmethylated CpG motifs that are generally absent, and mostly methylated in eukaryotic DNA [4]. The 2′-deoxyribose sugar backbone of DNA is also critical for recognition of oligodeoxynucleotides on naturally-formed phosphodiester backbones [5]. Bacterial and viral CpG-containing DNA (CpG-DNA) motifs, which can be mimicked by synthetic CpG-containing oligodeoxynucleotides (CpG-ODN), are recognized by TLR9 [6] and used as an adjuvant for treating infectious diseases, cancer, and allergies. TLR9 is most abundant in plasmacytoid dendritic cells and B cells and to lesser extent in monocytes, macrophages and neutrophils [7]–[9]. Like other nucleic-sensing TLRs, the intracellular localization and proteolytic maturation of TLR9 may reduce recognition of self-DNA [10].

CpG-DNA activation normally skews the host’s immune system to Th1-type responses *via* the MyD88-dependent pathway. In macrophages, bacterial DNA induces TNFα, IL-1, IL-6, IL-12, IFN-α/β, IFN-γ, and inducible nitric oxide synthase (iNOS) within hours, in pathways that may be direct or *via* other mediators [4]. For example, CpG-DNA directly induces IFN-β which promotes STAT1 phosphorylation and CXCL-10 (IP-10) production through the IFN-α/β receptor in an autocrine manner [11]. TLRs 3, 4 and 9 can also directly control expression of the anti-inflammatory cytokine IL-10 *via* the tumour necrosis factor receptor-associated factor 3 (TRAF3) pathway, indicating the importance of TLR signaling in restoration of immunological homeostasis [12]. Mechanisms restricting prolonged inflammation and limiting its damaging effects are just beginning to be unravelled and IL-10 signaling integrates several regulatory pathways [13].

S100 is a multigene family of 21 members of highly-conserved calcium-binding proteins [14]; S100A8 (also known as MRP8 or Calgranulin A) forms a stable complex with S100A9 (MRP9 or Calgranulin B) and these comprise the major calcium-binding proteins constitutively expressed in neutrophils and monocytes. Subsets of macrophages at inflammatory sites, but not normal tissue macrophages [15], [16] express the proteins and are regarded as a major source of S100A8 and S100A9 [17]. The S100A8/A9 complex (known as calprotectin) is antimicrobial and strongly expressed in lesions, and systemically, in patients with a variety of infections, and inhibits invasion and growth of a range of bacteria and fungi [18].

S100A8 is not always co-expressed with S100A9, particularly in elicited murine macrophages stimulated by LPS, Poly I:C, IFNγ or TNF [19] [20]. This, together with its chemotactic properties, which can cause mild transient leukocyte infiltration when injected into mice [21], originally suggested a proinflammatory role. However, corticosteroids [22], IL-10 and cAMP/PGE_2_ modulate LPS-induced mS100A8 expression [23] and S100A8 induction in response to TLR3 activation by Poly I:C or viral RNA, and to TLR4 by LPS, is IL-10-dependent [20]. Moreover, S100A8/A9 suppresses differentiation, antigen presentation, and release of inflammatory mediators such as IL-6, IL-12 and iNOS in dendritic cells [24]. This pattern of gene regulation in macrophages, and effects on dendritic cells, indicates anti-inflammatory properties. In keeping with this, we found that S100A8 scavenges oxidants, particularly peroxide, and hypochlorite (HOCl/OCl^−^) generated by the myeloperoxidase system [25] and may modulate redox in chronic inflammatory lesions such as in human atheroma [26] and human asthma [27]. Moreover, S100A8 is readily S-nitrosylated and this form suppresses leukocyte transmigration triggered by mast cell activation in the microcirculation [28]. Furthermore, suppression of ROS required for IgE-mediated signaling of antigen-sensitized mast cells may mediate the suppression of symptoms in acute asthma seen following S100A8 administration to murine lung [29]. S100A8 also reduces phosphorylation of p38 MAPK provoked by S100A9, and may modulate S100A9-associated phagocyte transmigration [30].

This is the first study to report the strong upregulation of an S100 protein by CpG-DNA in macrophages. S100A8 expression was dependent on two secondary gene products, IL-10 and PGE_2_ at the transcriptional level and involved multiple pathways, including MAPKs, PI3K, and JAK-STAT signaling. On the other hand, ERK1/2 activation, independent of IL-10 and PGE_2,_ was apparently required for direct S100A8 induction by CpG-DNA. The −178 to −34-bp region in the S100A8 promoter contained important *cis*-elements, STAT3, C/EBP, AP-1, Ets, and NF-1 binding sites that may contribute to its transcriptional activation. The induction pattern of S100A8 supports the proposal that S100A8 may regulate the magnitude of inflammatory responses and contribute to resolution.

## Materials and Methods

### Ethics statement

All protocols involving the use of human and murine macrophages were approved by the Human Research Ethics Committee and the Animal Care and Ethics Committee of the University of New South Wales, respectively. All participants provided their written informed consent to participate in this study. This consent procedure was approved by the Human Research Ethics Committee of the University of New South Wales.

### Materials

LPS was from *E. coli* O55:B5 (Sigma). Synthetic oligodeoxynucleotides (ODNs) were from Invitrogen and were resuspended in endotoxin-free Tris-EDTA buffer. The sequences of ODNs for positive activation of TLR9 at the indicated concentrations are: murine-specific CpG ODN 5′-TCCATGACGTTCCTGACGTT-3′ (CpG-1826, also referred to as CpG if not specifically indicated by types), human-specific CpG ODN 2216 5′-ggGGGACGATCGTCgggggg-3′ (CpG-2216, lowercase letters represent phosphorothioate linkage; capitals, phosphodiester linkage 3′ of the base). The sequence for non-nCpG control ODN 1982: 5′-TCCAGGACTTCTCTCAGGTT-3′ (nCpG). To prevent TLR9 and CpG colocalization in endosomal vesicles, thereby inhibiting TLR9 activation by its agonist, we pretreated cells with the inhibitory iCpG ODN 2088: 5′-TCCTGGCGGGGAAGT-3′ (iCpG) at the indicated concentrations for 30 min prior to stimulation with agonists. IL-10 and anti-mouse IL-10 mAb were from R&D systems. Cycloheximide (CHX), SB202190, U0126, JNK inhibitor II, LY294002, AG490, prostaglandin E_2_ (PGE_2_), indomethacin (Indo), and NS398 were from Calbiochem. Ruxolitinib was obtained from Selleck Chemicals. EP receptor antagonists SC-19220, AH-6809, and AH-2348 were from Sigma. PhosphoPlus Stat3 (Tyr705) antibody kit, including anti-phospho-Stat3 Ab, anti-Stat3 Ab, and untreated and IFNα-treated HeLa cell extracts, was from Cell Signaling.

### Macrophage culture

Culture media and reagents, including RPMI-1640 and Hank’s balanced salt solution, were either liquid or prepared from powder (Invitrogen) using Baxter water and filtered through 0.2 µm Zetapore filters (Cuno) to remove traces of endotoxin. Medium and mediators were only used if endotoxin levels were <20 pg/ml (chromogenic Limulus amebocyte assay; Associates of Cape Cod). Bovine calf serum (HyClone) was pretested for endotoxin content before purchase.

The murine macrophage-like cell line RAW 264.7 (ATCC, referred as RAW cells) was used within 15 passages after retrieval from frozen storage, and cultured at 37°C in a humidified atmosphere of 5% CO_2_ in air in RPMI-1640 supplemented with 100 U/ml penicillin, 100 µg/ml streptomycin (Invitrogen), and 10% heated (56°C, 30 min) bovine calf serum, referred to as complete medium (CM). RAW cells were passaged twice weekly in a 1∶40 split on Petri dishes (Falcon). Thioglycollate (TG)-elicited murine macrophages were obtained as described and cultured in RPMI 1640 with antibiotics supplemented with 2% BCS [23]. For mRNA measurements and protein analysis, macrophages were seeded into 24-well tissue culture plates (Nunc) at 1.5×10^5^ in 500 µl CM and cultured overnight before stimulation.

Human monocyte-derived macrophages were prepared as described [31]. Briefly, PBMCs isolated from peripheral blood of health subjects using Ficol-paque plus (GE Healthcare) were dispensed into 24-well Costar plates (2.5×10^6^/well) in RPMI-1640 containing 10% heated (56°C, 30 min) autologous serum, 2 mM L-glutamine, penicillin/streptomycin, and 10 ng/ml M-CSF and incubated at 37°C in 5% CO_2_ in air for 3 days. Non-adherent cells were gently rinsed off with warm RPMI-1640 and adherent cells cultured in RPMI-1640 with 10% autologous serum and antibiotics for another 4 days. For experiments using pathway inhibitors, cells were pretreated with inhibitors for 20 min, then untreated or stimulated for the indicated time.

### DNA manipulation

*E. coli* DNA, *M. luteus* DNA, and human placental DNAs (Sigma) were further purified by two-step CsCl ultracentrifugation [32]. DNA digestion was performed using RNase-free Turbo DNase (Ambion) at 2 U/µg DNA in buffer supplied with the enzyme, for 2 h at 37°C. Endotoxin levels in all DNA preparations were <2 pg/µg DNA, assayed by the *Limulus* amebocyte lysate assay.

### RNA isolation and quantitative RT-PCR analysis

RNA preparation and real time RT-PCR were performed essentially as described [14]. Briefly, 1.0 µg was treated with Turbo DNase to eliminate DNA and then converted to cDNA using SuperScript VILO cDNA Synthesis Kit (Life technology) according to the manufacturer’s instructions. Real-time PCR was performed with LightCycler 480 SYBR Green I Master (Roche) on a LightCycler 480 Real-Time PCR System (Roche) under standard cycle conditions. Relative quantities of mRNA in duplicate samples were obtained using the LightCycler 480 Software 1.5 and the Efficiency-Method and normalized against hypoxanthine-guanine phosphoribosyltransferase (HPRT) or β-actin as endogenous controls, there is no difference in terms of fold changes using either endogenous controls. Unless otherwise stated, data represent the fold changes of mRNA relative to unstimulated samples; means ± SD of 3 separate experiments are given. The PCR primer sequences used for S100A8, S100A9 and HPRT were as described [14]. Other primer sequences were IL-10 (forward: GGTTGCCAAGCCTTATCGGA, reverse: ACCTGCTCCACTGCCTTGCT), COX-2 (forward: TGAGCAACTATTCCAAACCAGC, reverse:GCACGTAGTCTTCGATCACTATC), C/EBPβ (forward: TGATGCAATCCGGATCAA, reverse: ACACGTGTGTTGCGTAGTCC) and β-actin (forward: AGTGTGACGTTGACATCCGTA, reverse: GCCAGAGCAGTAATCTCCTTCT).

### S100A8 and IL-10 measurements

S100A8 levels in culture supernates from untreated or stimulated RAW 264.7 cells were quantified by ELISA as described [20]. IL-10 was quantitated using the IL-10-specific DuoSet ELISA kit (R&D Systems; Minneapolis) according to manufacturer’s instructions.

### Western blotting

RAW cells were incubated in CM ± CpG-1826 or IL-10 for the indicated times. Following lysis (M-PER Extraction Reagent, Pierce Biotechnology), total protein content was determined by the Bradford assay (Bio-Rad). Protein (30 µg) was separated by 10% SDS-PAGE, and resolved proteins transferred to PVDF membranes (Millipore). Blots were probed with anti-phospho-Stat3 (Tyr 705) or -Stat3 Abs, detected with HRP-conjugated anti-rabbit Ab and visualized by ECL (Millipore). Lysates of HeLa cells treated with IFNα (5 min) were used as a positive control for detection of phosphorylated STAT3.

### Luciferase reporter gene assays

Constructs of S100A8 luciferase reporter plasmids and transient transfection of RAW cells were described elsewhere [23]. Briefly, cells were seeded into 24 well plates (Nunc, 0.3×10^5^ per well) 2 days before transfection. S100 promoter luciferase reporter constructs or pGL-basic and pGL-promoter control plasmids (Promega, 0.3 µg) and 0.05 µg reference plasmid (pRL-TK; Promega) were co-transfected in the presence of 300 µg/ml DEAE-dextran (Sigma-Aldrich). For Stat3 plasmid co-transfection, cells were transfected with 0.2 µg S100A8 promoter construct (−178/465) in combination with 0.8 µg vector pcDNA3.1 (Invitrogen) or the dominant-positive Stat-3C cloned into pcDNA3.1, or vector pIRES-EGFP, or the dominant-negative Stat-3β cloned into pIRES-EGFP, which are previously described [33]. All Stat3 and control vectors were kindly provided by Dr. Hua Yu (Beckman Research Institute, City of Hope, California, USA). After recovery for 24 h, fresh medium was replaced and cells stimulated for 20 h with the agents indicated, then firefly and *Renella* luciferase activities analysed using the dual luciferase assay kit (Promega). Results are expressed as means ± SD of luciferase activities from 3 separate experiments.

### Chromatin immunoprecipitation (ChIP) assays

RAW cells (2×10^6^) in 3 ml CM in 6-well plates were cultured for 24 h before stimulation. Assays were performed essentially as described [20]. Anti-STAT3 polyclonal IgG and control IgG (Santa Cruz Biotechnology) were used at 2 µg for each reaction.

### Statistical analysis

Data for statistical analyses are expressed as means ± SD of at least 3 independent experiments. Statistical comparisons between groups were assessed by Student’s *t*-test, or between multiple groups by one-way ANOVA with post-analysis. Statistical significance was defined as *p*<0.05.

## Results

### CpG motifs in bacterial DNA induce S100A8, but not S100A9

**Figure 1:**
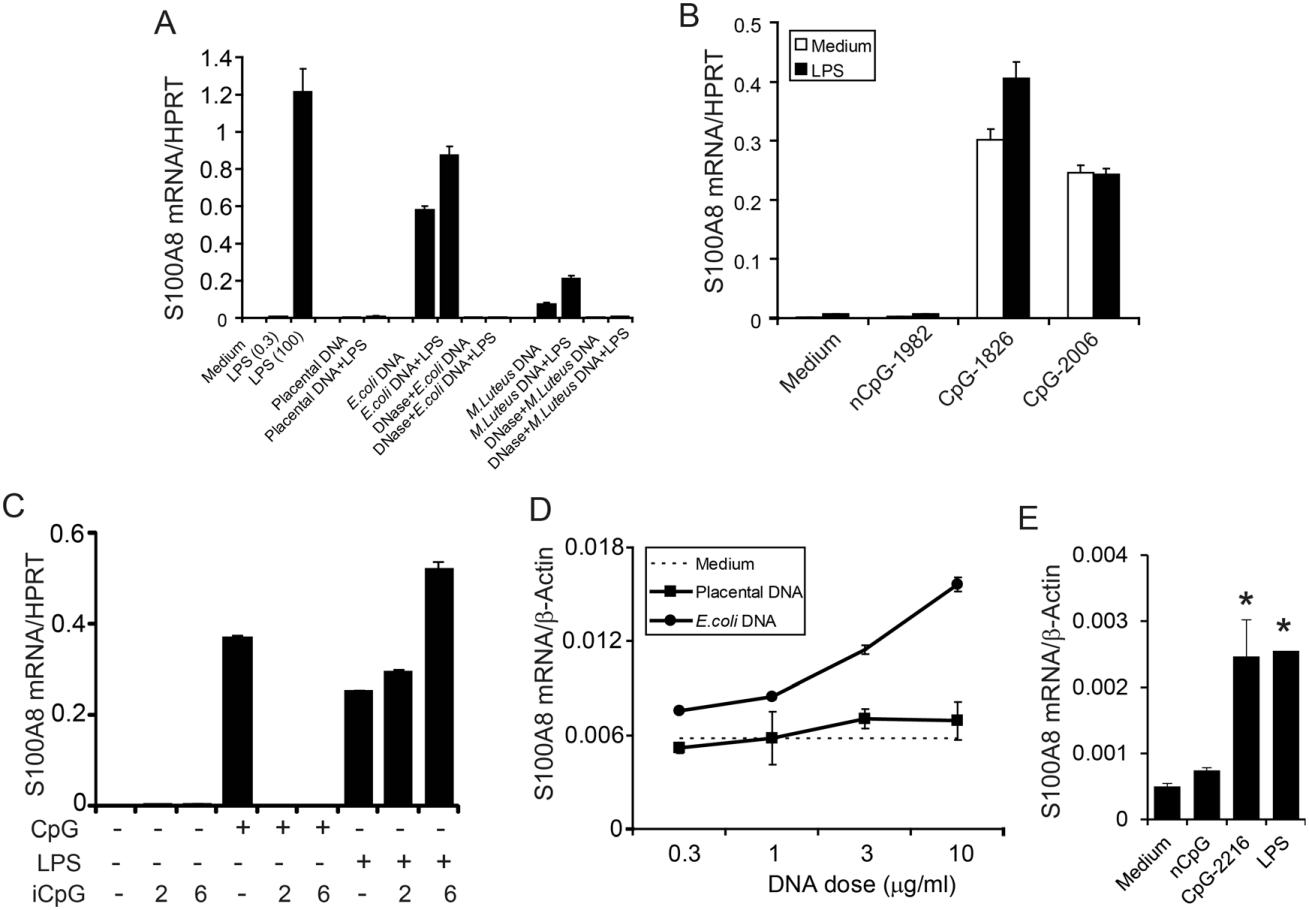
Induction of S100A8 mRNA in macrophages by *E. coli* DNA, *M. luteus* DNA, and synthetic CpG-ONDs. (A) RAW 264.7 macrophages were incubated with *E. coli* DNA (3 µg/ml) or *M. luteus* DNA (3 µg/ml) ±0.3 ng/ml LPS. Controls include placental DNA (3 µg/ml) ± LPS (0.3 ng/ml), Turbo DNase-treated *E. coli* DNA or *M. luteus* DNA ± LPS (0.3 ng/ml). RAW cells were also stimulated with two doses of LPS alone as positive controls (0.3 or 100 ng/ml). (B) RAW cells were untreated (medium) or treated with a synthetic non-CpG-containing OND 1982 (nCpG), a murine-oriented CpG-containing ODN (CpG-1826), or a human-oriented CpG-containing ODN (CpG-2006) (all 3 µM final concentration) ± LPS (0.3 ng/ml). (C) medium control, CpG-1826 (3 µM) or LPS (20 ng/ml) were co-stimulated with inhibitory CpG ODN 2088 (iCpG) 2 or 6 µM. (D) Elicited murine macrophages were untreated or stimulated with human placental DNA or *E. coli* DNA at the indicated concentrations. (E) Human monocyte-derived macrophages stimulated with 3 µM of nCpG or CpG-2216; LPS (20 ng/ml) was positive control. Combined data from 3 independent experiments are presented as the mean ± SD (*, p<0.05). All treatments were for 24 h before cell harvest. Total RNAs were extracted and S100A8 mRNA quantitated by real-time RT-PCR and normalized to the house-keeping genes (murine HPRT was used as endogenous control for A, B and C; murine β-actin for D; human β-actin for E). All data, except E, are represented as means (relative to the HPRT or actin mRNA levels) ± SD of duplicate measurements and are representative of at least 3 experiments.

The TLR4 ligand, LPS, strongly induces S100A8, but not S100A9, in murine macrophages [34]. Because human S100A8 and S100A9 are considered markers of specific types of human inflammatory macrophages [16], and because CpG-containing DNA activates macrophages via TLR9 ligation, we tested whether the S100 genes were induced. RAW 264.7 macrophages were exposed to various DNA products ± a predetermined subthreshold concentration of LPS (0.3 ng/ml). *E. coli* DNA directly induced S100A8 mRNA ∼400-fold above basal levels compared to the ∼700-fold induction by an optimized dose of LPS (100 ng/ml; Fig. 1A). DNA from the Gram-positive organism *M. luteus* increased S100A8 mRNA 50-fold. Simultaneous treatment with *E. coli* DNA and a suboptimal LPS (0.3 ng/ml) dose used to identify synergy, only increased S100A8 induction <1.4-fold. In contrast to the bacterial DNAs, placental DNA, which contains highly methylated CpG dinucleotides, did not induce S100A8 mRNA either alone, or with subthreshold levels of LPS, confirming that activation was dependent upon non-methylated CpG motifs. This was confirmed using the synthetic non-methylated CpG-containing murine-specific ODN 1826, or the human-oriented ODN 2006, although mRNA levels were lower (Fig. 1B), again with little synergy with LPS.

In contrast to the immune-reactive ODNs, non-CpG-containing ODN 1982 did not influence S100A8 gene expression (Fig. 1B). Importantly, when bacterial DNA was treated with DNase (Fig. 1A) or CpG-ODN co-incubated with inhibitory iCpG (Fig. 1C), responses were ablated. Conversely, iCpG did not affect LPS-induced responses, confirming the specificities of each interaction. S100A9 is co-expressed with S100A8 in neutrophils and in human monocytes, but was below the detection limit when cells were stimulated with CpG-containing DNA (table S1, bone marrow cells were used as positive controls).

*E. coli* DNA also induced S100A8 mRNA in elicited murine macrophages in a dose-dependent manner whereas human placental DNA had no effect (Fig. 1D). In human monocyte-derived macrophages, S100A8 induction by type A CpG-OND (2216), but not by nGpG, confirming a specific response to the type A CpG motif, and was similar to levels generated with a pre-optimized dose of 10 ng LPS.

**Figure 2:**
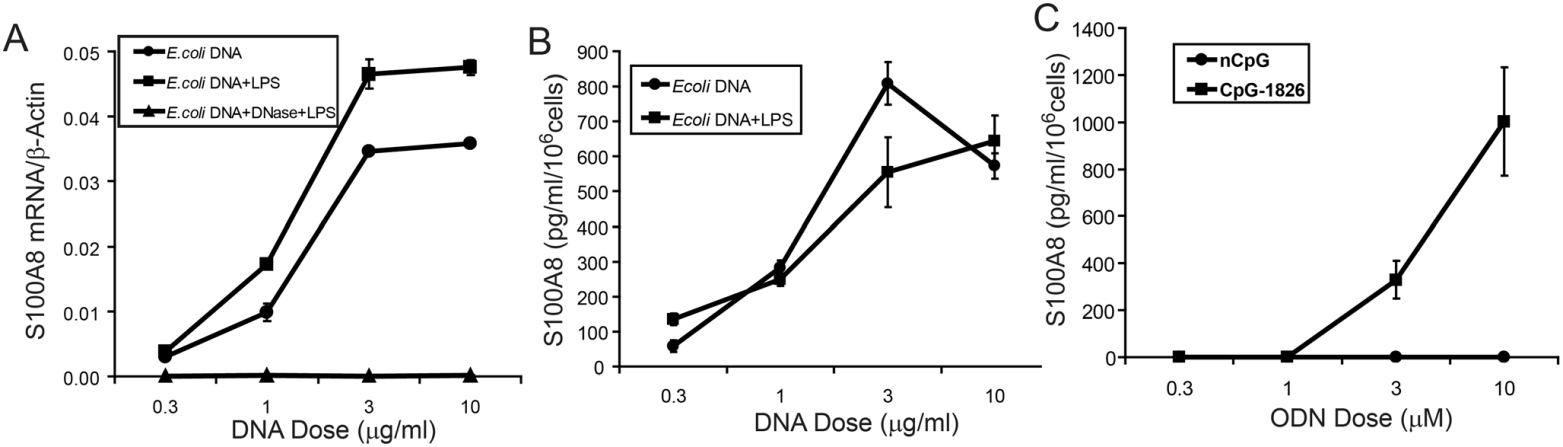
Induction of S100A8 by CpG-DNA is dose-dependent. (A) RAW 264.7 macrophages were incubated with *E. coli* DNA at the indicated concentrations ±0.3 ng/ml LPS for 24 h before harvesting and (A) mRNAs quantitated by qRT-PCR with different doses of DNase-treated *E. coli* DNA ± LPS (0.3 ng/ml) as control. (B) S100A8 levels in culture supernates from (A) were quantitated by ELISA. Other controls included various concentrations of placental DNA ± LPS (0.3 ng/ml) or DNase-treated *E. coli* DNA, but mRNA levels were same as control and proteins were below the detectable level (not shown). (C) S100A8 in supernates from RAW cells treated with synthetic nCpG-1982 or CpG-1826 at the indicated concentrations was quantitated. QRT-PCR data represent means (relative to the γ-actin mRNA levels) ± SD of duplicates and are representative of 3 experiments. ELISA data are means ± SD of duplicate samples from 2 separate experiments.

S100A8 mRNA induction by *E. coli* DNA was dose-dependent between 0.3–3 µg/ml, with little increase with higher doses; LPS only slightly increased mRNA levels over the entire dose range (Fig. 2A). Neither DNase-treated DNA (Fig. 2A) nor human DNA (not shown) was active at these doses. S100A8 is secreted by stimulated macrophages [23]. Fig. 2B shows that S100A8 increased from 57.9±16 pg/ml/10^6^ cells stimulated with 0.3 µg/ml *E. coli* DNA to 808.9±61 pg/ml/10^6^ cells with 3 µg/ml DNA; LPS only slightly (1.4-fold) increased amounts. Consistent with mRNA expression, neither human DNA nor DNase-treated *E. coli* DNA generated S100A8 (not shown). Supernates from RAW cells treated with 10 µg/ml CpG-containing ODN 1826 contained high amounts of S100A8 whereas control ODN 1982 failed to generate detectable secreted protein (Fig. 2C). These experiments confirmed that DNA-containing CpG-dinucleotides strongly induced S100A8 in a dose-dependent manner.

### Induction of S100A8 by *E. coli* DNA requires IL-10 and PGE_2_

**Figure 3:**
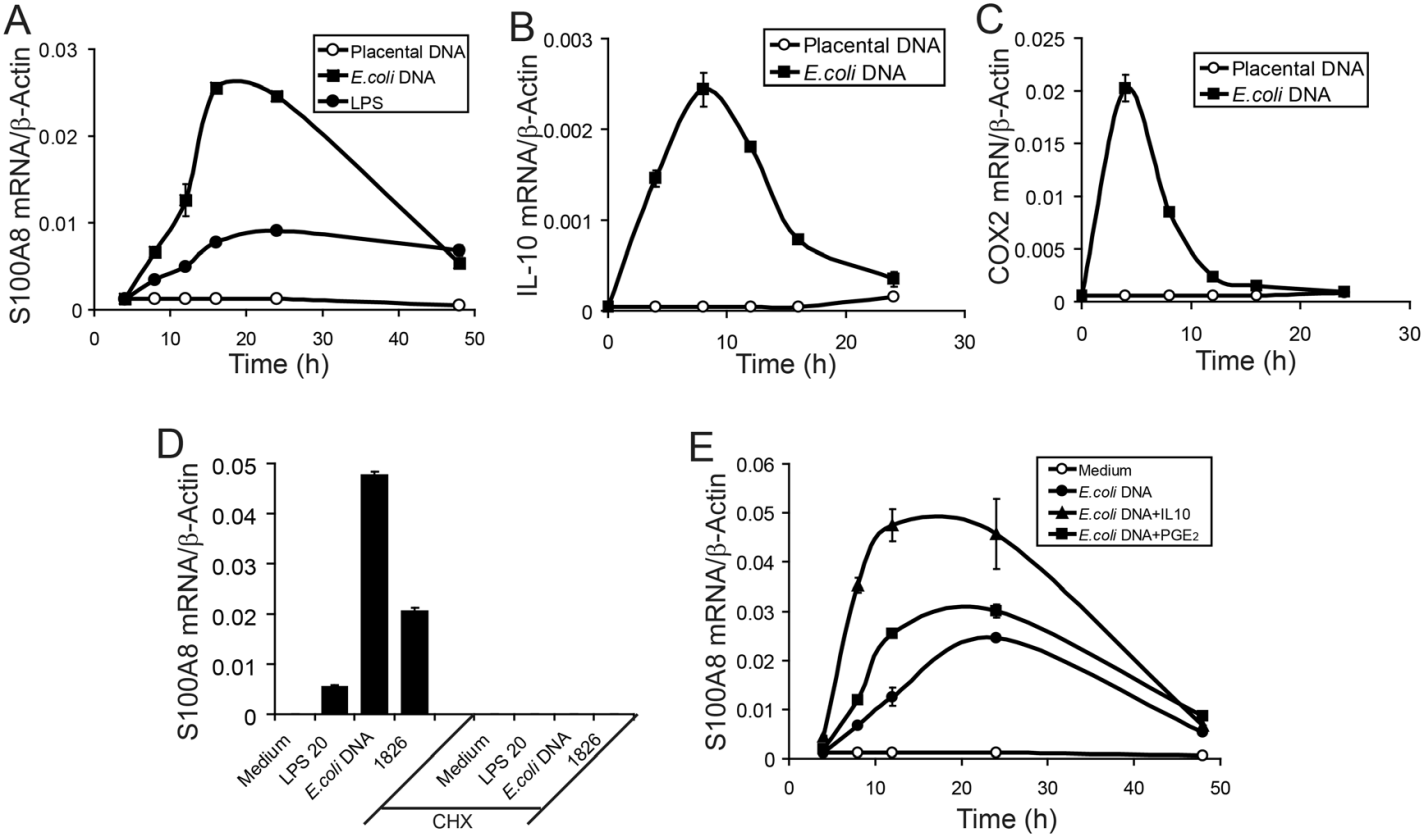
Induction of S100A8 in macrophages by *E. coli* DNA is late and requires newly synthesis proteins. (A–C) RAW264.7 macrophages were treated with 3 µg/ml placental DNA, 3 µg/ml *E. coli* DNA, or 20 ng/ml LPS for the indicated times. S100A8 (A), IL-10 (B), or COX-2 (C) mRNAs quantitated by qRT-PCR. (D) Effects of CHX (2 µg/ml) on LPS (20 ng/ml), *E. coli* DNA (3 µg/ml), or CpG-containing ODN (1826, 3 µM)-induced S100A8 mRNA expression. (E) Cells incubated with *E. coli* DNA (3 µg/ml) ± IL-10 (2 ng/ml) or ± PGE_2_ (10 µM), harvested at the indicated time points and mRNAs quantitated. Data represent means ± SD of duplicates and representative of 3 experiments.

CpG-DNA induces IL-10 and COX-2, an inducible rate-limiting enzyme that converts arachidonic acid to PGE_2_. We compared kinetics of expression of these genes, with S100A8 induction. Fig. 3A shows that *E. coli* DNA, but not placental DNA, increased S100A8 mRNA levels from 8 h, was optimal at 16–24 h, and declined to 15% of peak levels by 48 h. Similarly, S100A8 mRNA induction by LPS was maximal at ∼24 h, but relatively stable over 48 h [23]. In contrast, induction of IL-10 (Fig. 3B) and COX-2 (Fig. 3C) mRNAs by *E. coli* DNA was more rapid, peaking at 8 h and 4 h respectively, returning to baseline by 24 h. Placental DNA induced neither gene.

Cycloheximide (CHX) pretreatment completely blocked S100A8 mRNA induction in RAW cells treated with *E. coli* DNA, CpG-1826 or LPS (Fig. 3D), confirming a secondary event dependent on new protein(s) synthesis. IL-10 and PGE_2_ generally inhibit induction of proinflammatory mediators. These do not directly induce S100A8 in murine macrophages, but potentiate its induction by LPS [23]. Both mediators enhanced induction by *E. coli* DNA (Fig. 3E) and promoted earlier S100A8 mRNA induction. At 8 h, IL-10 increased S100A8 mRNA levels 6-fold and at 12 h, ∼4-fold. PGE_2_ increased S100A8 mRNA ∼2-fold at these time points.

**Figure 4:**
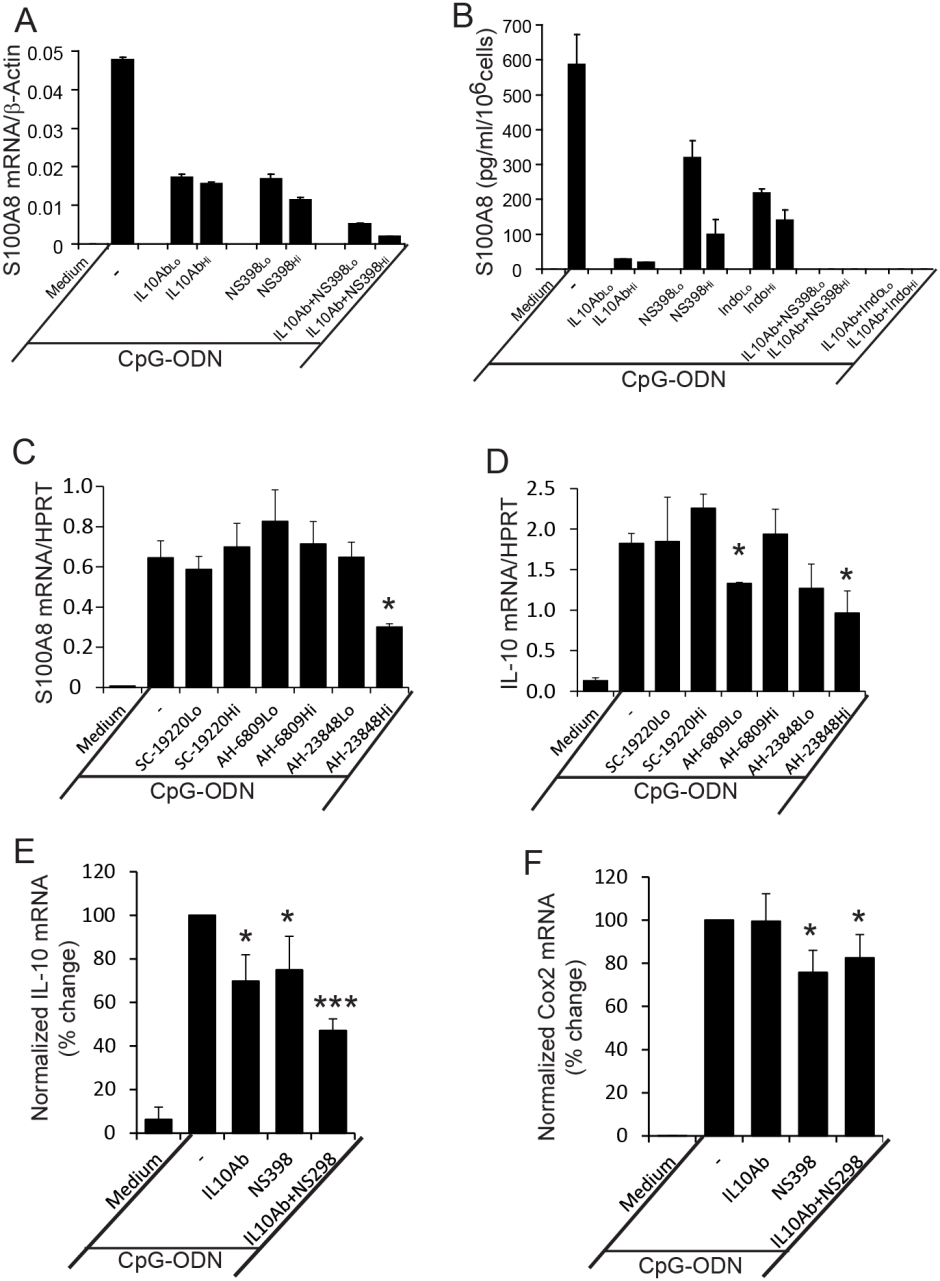
Induction of S100A8 in macrophages by CpG-ODN requires both IL-10 and PGE_2_/EP4 dual signaling pathways. (A and B) RAW cells were incubated with CpG-ODN (3 µM) for 20 h in the presence/absence of two doses (Lo: low, 1 ng/ml; Hi: high, 10 ng/ml) of IL-10 Ab and/or COX inhibitors (NS389: Lo, 5 µM, Hi, 50 µM; Indomethacin (Indo): Lo, 2 µM; Hi, 20 µM). Cells and supernates were harvested, and S100A8 mRNA (A) or protein (B) quantitated. Data represent means ± SD of duplicates and representative of 3 experiments. (C and D) RAW cells were incubated with CpG-ODN (3 µM) in the presence/absence of two doses (Lo, 1 µM; Hi, 10 µM) of antagonists for EP1 (SC-19220), EP2 (AH-6809), and EP4 (AH-23848) for 8 (C) and 20 h (D). IL-10 (C) and S100A8 mRNA (D) quantitated by qRT-PCR. (E and F) RAW cells were untreated or treated with CpG-ODN (3 µM) for 6 h in the presence/absence of anti-IL-10 Ab (5 ng/ml) and/or NS389 (25 µM), and IL-10 (E) and Cox2 (F) mRNA quantitated by qRT-PCR, For C–F, data represent means ± SD of −5 separate experiments. *P<0.05, ***P<0.001 of differences between samples treated with CpG-ODN alone and CpG-ODN plus various inhibitors.

The requirement for IL-10 in S100A8 induction, was confirmed using a neutralizing anti-IL-10 mAb which decreased CpG-DNA-induced S100A8 mRNA by 65% (Fig. 4A), and protein levels by 94% (Fig. 4B); 10-fold more mAb only marginally increased inhibition. The specific COX-2 inhibitor (NS389) suppressed S100A8 mRNA induction to the same extent (Fig. 4A), although suppression of protein production required a higher dose to achieve 83% reduction (Fig. 4B). Indomethacin, a general COX-1 and COX-2 inhibitor, also inhibited S100A8 protein production (Fig. 4B). Importantly, effects of IL-10 and COX-2 inhibitors were synergistic, and when combined, S100A8 mRNA levels decreased almost to baseline (Fig. 4A), with no secreted S100A8 detected (Fig. 4B). Thus, IL-10 and/or PGE_2_ generated by TLR9 ligation may act together or independently to promote S100A8 induction in macrophages.

PGE_2_ exerts its action through G protein-coupled E prostanoid (EP) receptors 1–4. Using antagonists for EP1, 2 and 4 at two doses, we found that the EP4 antagonist (AH-2348; 10 µM) significantly suppressed S100A8 mRNA induction by CpG-ODN by 54% (Fig. 4C). Interestingly, IL-10 mRNA induction at 8 h was also suppressed by 48%. The EP2 antagonist also significantly inhibited IL-10 mRNA by 28% (Fig. 4D). Taken together, S100A8 mRNA induction in macrophages by TLR9 ligands required IL-10 and PGE_2_ activation via the IL-10 receptor and EP4, respectively.

We also examined the cross regulation between IL-10 and PGE_2_ pathways. Similar to S100A8 induction, inhibition of IL-10 or COX-2 pathways reduced IL-10 mRNA expression by ∼25%, and more evident suppression (43%) was observed when both pathways were inhibited (Fig. 4E). CpG-DNA induced COX-2 mRNA was also self-regulated, but not by IL-10 production (Fig. 4F). Results indicate self- and/or cross-regulations of IL-10 and PGE_2_ pathways, and further complicate the late induction of S100A8.

### Role of MAPKs, PI3K and JAK2 in CpG-DNA-mediated S100A8 induction

**Figure 5:**
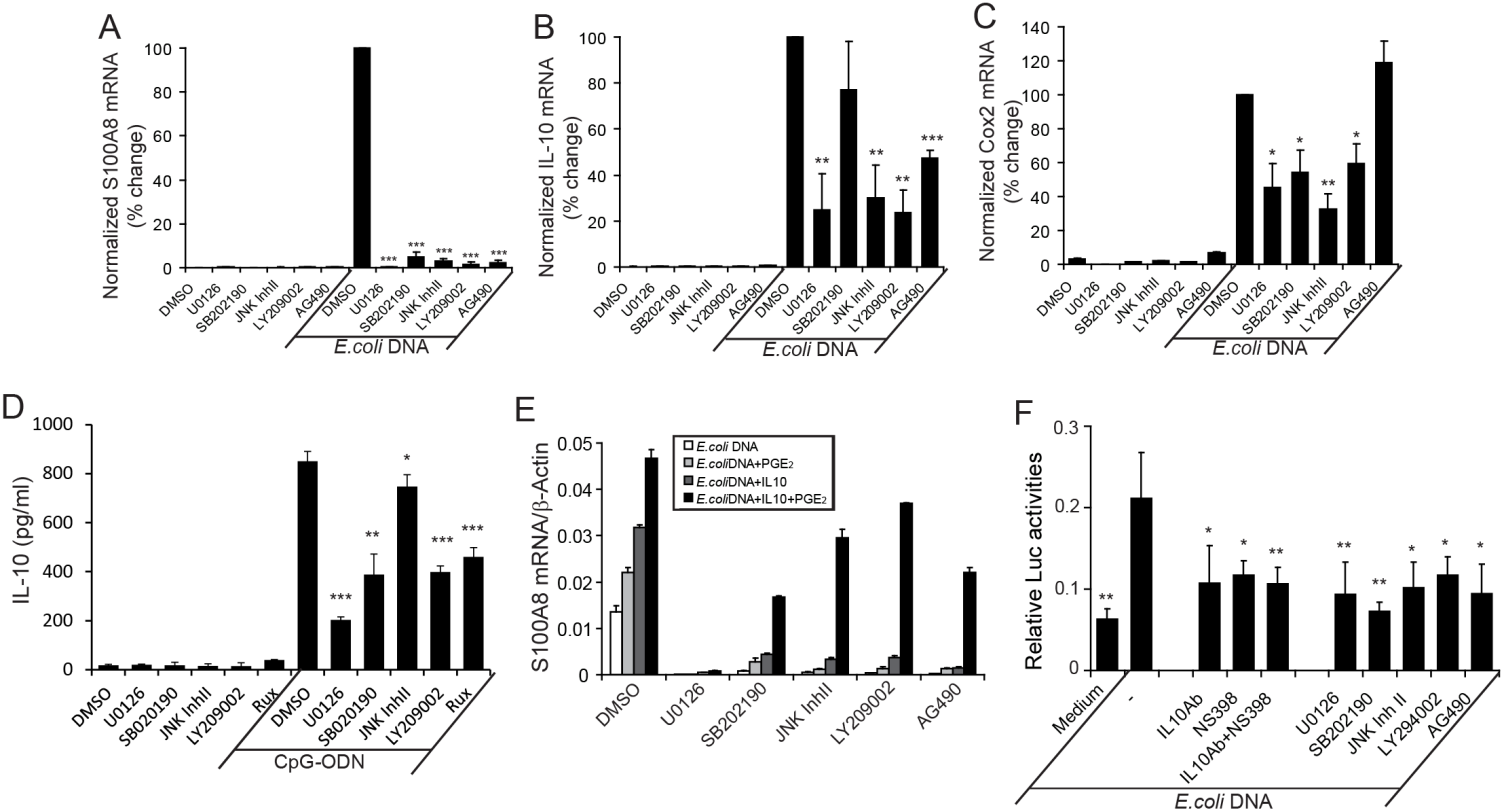
Contribution of MAPKs, PI3K, and JAK2 to *E. coli*-DNA-mediated S100A8, IL-10 and COX-2 mRNA induction. (A–C) RAW 264.7 cells were pretreated with DMSO (vehicle control), U0126 (2.5 µM), SB202190 (2.5 µM), JNK inhibitor (5 µM), LY209002 (10 µM), or AG490 (20 ng/ml) for 20 min then untreated or stimulated with *E. coli* DNA (3 µg/ml) for 20 h (A), or 4 h (B, C). mRNA for S100A8 (A), IL-10 (B) or COX-2 (C) was quantitated using qRT-PCR. (D) RAW cells were treated using same conditions as in (A), except that AG490 was replaced by Ruxolitinib (0.5 µM); IL-10 in supernatants were quantitated by ELISA. (E) RAW cells were treated with inhibitors for 20 min, stimulated with *E. coli* DNA for 30 min, and then ± PGE_2_ (10 µM) or IL-10 (5 ng/ml) added and cells cultured for 20 h. S100A8 mRNA levels were quantitated; endogenous control was γ-actin. Data represent % S100A8 mRNA relative to *E. coli* DNA-stimulated samples without inhibitors ± SD of 3 separate experiments. (F) RAW 264.7 cells were transiently co-transfected with the S100A8 promoter (–178/465 bp) luciferase reporter construct and pRL-TK-luciferase control plasmid and pretreated with various MAPK and COX-2 inhibitors, or anti-IL-10 mAb for 20 min before adding *E. coli* DNA (3 µg/ml) for 16 h. Normalized S100A8 promoter-driven luciferase activities in cell extracts were analysed. Except for (E), data represent means ± SD of 3–4 separate experiments. *P<0.05, **P<0.01, ***P<0.001 of differences between *E. coli* DNA treatment alone and samples treated with *E. coli* DNA plus various inhibitors.

In RAW 264.7 cells, CpG-DNA activates the three MAPK pathways: ERK, p38, and JNK [35]. Using the concentrations shown to specifically inhibit particular MAPKs in these cells [35], we found that these MAPK pathways were involved in S100A8 induction. U0126, SB202190, and JNK inhibitor II (inhibit ERK, p38, and JNK respectively) all abolished *E. coli* DNA-induced S100A8 mRNA by >95% when measured at the time of maximal expression (20 h). These inhibitors did not affect baseline S100A8 mRNA levels seen with vehicle (DMSO) in the absence of CpG-DNA (Fig. 5A). Effects of these inhibitors on IL-10 and COX-2 mRNA induction at 4 h, when both genes were strongly induced (Figs 5B, 5C), and secreted IL-10 protein at 20 h (Fig. 5D) indicated involvement of ERK and JNK in induction of both genes. p38 was significantly involved in IL-10 protein induction, but at mRNA levels, indicating its post-transcriptional role. Unlike S100A8, suppressions of IL-10 or COX-2 were only partial (compare Fig. 5B, 5C with 5A).

PI3K is also involved in TLR-mediated activation [36]. LY294002, a specific PI3K inhibitor, totally abolished S100A8 mRNA induction by CpG-DNA (Fig. 5A) and significantly reduced IL-10 and COX-2 mRNAs by 76% and 48%, respectively (Fig. 5B and 5C). Activation by IL-10 receptors (but not TLR) involves the JAK-STAT pathway. AG490, a JAK-2 inhibitor, reduced CpG-DNA- induced S100A8 mRNA by 97% (Fig. 5A) and IL-10 mRNA by ∼53% (Fig. 5B), but did not affect COX-2 mRNA levels (Fig. 5C). Similarly, using recently developed, more specific JAK-2 inhibitor Ruxolitinib (Rux), CpG-DNA-induced S100A8 and IL-10 mRNAs were suppressed by 93% and 62% respectively (fig. S1C and 1A), with no significant reduction in COX-2 mRNA (fig. S1B). Thus, S100A8 induction by CpG-DNA appears complex, requiring multiple pathways, including MAPKs, PI3K, and JAK-STAT activation.

As S100A8 induction by CpG-DNA was indirectly modulated by IL-10 and COX-2-mediated events via the MAPK and PI3K pathways, we examined whether exogenous IL-10 or PGE_2_ or their combination, facilitated S100A8 production in the presence of inhibitors. Fig. 5E shows that PGE_2_ and IL-10 increased CpG-DNA-induced S100A8 mRNA 1.6- and 2.3-fold respectively; a 3.5-fold increase was apparent when combined. S100A8 mRNA was suppressed by >95% with the P38, JNK, PI3K, and JAK2 inhibitors (Figs 5A and 5E). Exogenous IL-10 or PGE_2_ only marginally increased S100A8 mRNA levels generated in the presence of inhibitors, compared to CpG-DNA stimulation in the absence of inhibitors. However when combined, IL-10 and PGE_2_ completely rescued S100A8 mRNA induction (1.3, 2.2, 2.7, and 1.6-fold above CpG-DNA-treated control, in cells treated with SB202190, JNK inhibitor II, LY209002, or AG490, respectively). Combined addition of IL-10 and PGE_2_ also significantly induced S100A8 mRNA compared to individual components when JAK pathway was specifically inhibited by Rux (fig. S1C). Thus, suppression of S100A8 production by these inhibitors may be a direct consequence of reduced IL-10 and/or PGE_2_ production by inhibitor-treated cells. In marked contrast, the ERK inhibitor (U0126) strongly suppressed S100A8 mRNA induction but this was not influenced by IL-10 or PGE_2_ addition (Fig. 5E). Thus ERK1/2 activation appeared to be directly involved in S100A8 induction by CpG-DNA, independent of IL-10 and PGE_2_.

**Figure 6:**
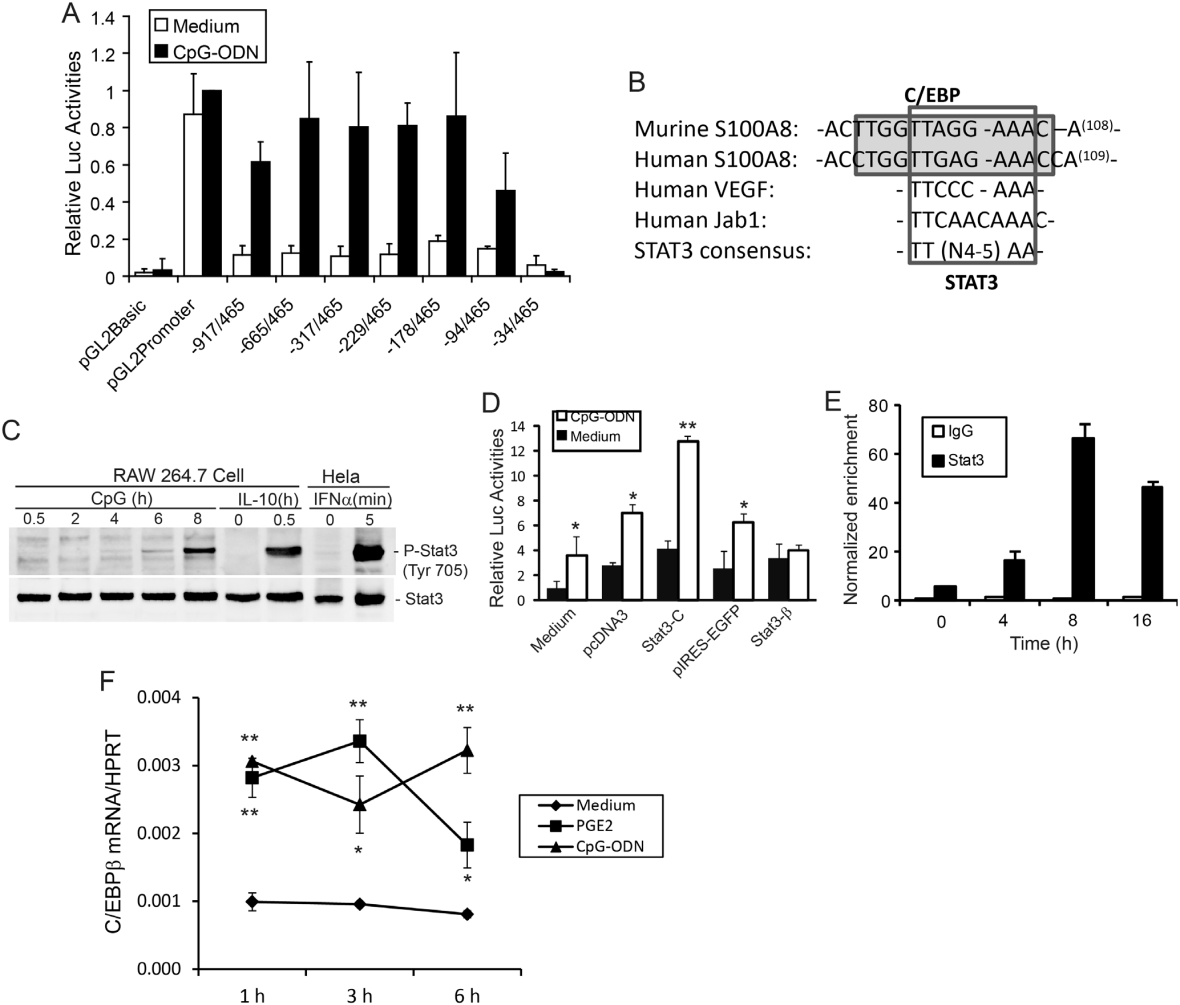
STAT3 is a critical transcription factor in CpG-DNA-induced S100A8 expression. (A) RAW 264.7 cells were transiently co-transfected with pRL-TK-luciferase and full-length (−917/465) or a series of 5′-deleted S100A8 promoter-luciferase reporters for 24 h. pGL-basic and pGL-promoter plasmids were transfected as controls. Transfected cells were either left untreated (open bars) or stimulated with CpG-ODN (3 µM, solid bars) for 16 h. Luciferase activities in cell extracts were analysed; data represent means (% luciferase activity of pGL-promoter luciferase reporter in cells stimulated with CpG-ODN) ± SD of 3 separate experiments. (B) A potential STAT3-binding element overlaps the C/EBP binding site in both murine and human S100A8 promoter (shaded). The matched bases of STAT3 binding sites are compared with verified binding sites in the VEGF and Jab1 promoters. (C) RAW cells were treated with CpG-ODN (3 µM) for the indicated times (h) before harvest, then proteins in lysates separated by SDS-PAGE. Western blotting used a phosphor-specific anti-STAT3 antibody; stripped blots were re-probed with anti-STAT3 antibody. RAW cells treated with IL-10 for 30 min or IFNα-treated Hela (5 min) were positive controls. Results are representative of 3 experiments. (D) RAW 264.7 cells were transiently co-transfected with the S100A8 promoter (−178/465 bp) luciferase reporter construct and pRL-TK-luciferase control plasmid, with either empty vectors, dominant-positive (Stat3-C), or dominant-negative (Stat3-β) STAT3 constructs for 24 h. Cells were then stimulated with (□) or without (▪) CpG-ODN (3 µM) for 16 h prior to performing dual-luciferase assays. Results are mean ± SD of 3 independent experiments. *P<0.05 or **P<0.01 of differences with or without CpG-ODN treatments. (E) Time course of ChIP assays indicated binding of STAT3 to the S100A8 promoter in RAW cells stimulated with CpG-ODN (3 µM) (▪). The same region was not enriched by IgG control (□). (F) RAW cells were untreated or treated with 10 µM PGE_2_ or 3 µM CpG-ODN for the indicated time and C/EBPβ mRNA quantitated by qRT-PCR. Results are means ± SD of 3 independent experiments. *P<0.05 or **P<0.01 of differences compared to medium control.

Using the smallest construct identified in luciferase reporter assays (Fig. 6A and discussed below) that preserved the full response to CpG-DNA (–178/465), we next analysed the dependence of transcriptional regulation of S100A8 on IL-10 and PGE_2_ via the MAPK, PI3K, and JAK pathways. Anti-IL-10 mAb or the COX-2 inhibitor reduced relative CpG-DNA-induced luciferase activities by ∼50% (Fig. 5F), confirming their involvement at the transcriptional level, in a region largely confined to the −178 to −34 bp region of S100A8 promoter. P38, JNK, PI3K, and JAK inhibitors also significantly reduced luciferase activities driven by this region in response to CpG-DNA. The ERK1/2 pathway inhibitor U0126 reduced luciferase activity by 56%, indicating that this region of the promoter also contributed to direct transcription of the S100A8 gene via ERK1/2 activation (Fig. 5F).

Taken together, we propose that CpG-DNA induction of S100A8 at the transcriptional level is dependent on generation of IL-10 and COX-2/PGE_2_ via p38, JNK, PI3K and JAK-STAT pathways, together with direct signaling through ERK1/2.

### STAT3 mediates induction of S100A8 by CpG-DNA

Gene induction by CpG-DNA involves alteration of transcriptional activities and/or transcript stability [6]. To confirm S100A8 induction at the transcriptional level; and to locate likely response elements in the promoter region 5′ to the transcription start site of the gene, a series of 5′ deletion fragments linked to a luciferase reporter gene were transiently transfected into RAW cells. A region ∼1.4 kb 5′ flanking the translation start site of the murine S100A8 gene, including a 917 bp 5′ region flanking the transcription start site, untranslated first exon and intron, and the untranslated region of the second exon, served as the full-length promoter (−917/465). Fig. 6A shows that CpG-DNA increased the activity of this full-length reporter ∼6-fold above basal levels. Luciferase activities of a series of 5′ deletions from −917 bp to −178 bp (constructs −665/465, −317/465, −229/465, and −178/465) were similarly elevated, confirming induction at transcription; the region −917 bp to −178 bp did not contribute substantially. Further deletion of 84-bp from the 5′ end of −178/465 (construct −94/465), a region containing one Ets, two STAT, and three C/EBP binding sites (TRANSFAC database), reduced CpG-DNA-induced S100A8 promoter-luciferase activity by ∼47%. These C/EBP binding sites are highly conserved between murine and human (Fig. 6B) and C/EBPβ mRNA was rapidly induced by CpG-DNA or PGE_2_ at 1 h and sustained to 6 h (Fig. 6F), indicating the critical role of this transcription factor in S100A8 induction. Deletion of an additional 60 bp (−34/465), removing an NF-1 and an Ets site, but retaining the TATA box, completely abolished reporter activity (Fig. 6A). Thus, the −178 to −34-bp region in the S100A8 promoter contained important *cis*-elements, including C/EBP, STAT, Ets, and NF-1 binding sites that may contribute to S100A8 gene induction by CpG-DNA.

IL-10 signaling is dependent on STAT3 to mediate anti-inflammatory responses [37]; STAT3 is also involved in transcription of S100A8 and S100A9 in myeloid-derived suppressor cells (MDSC) [38] but precise mechanisms are unclear. We next tested whether STAT3 contributed to transcriptional activation of the defined promoter region. By searching for potential binding sites with the consensus sequences for STAT3, TT(N4)AA or TT(N5)AA [39], we identified a perfect candidate at position −117 upstream of the transcription initiation site in the murine S100A8 promoter, which is 100% conserved in the human S100A8 promoter. This site closely resembles a previously-defined STAT3-binding site within the VEGF and Jab1 promoters [33], [40] (Fig. 6B). Functionally-active phospho-STAT3 was generated 4 h after stimulating RAW cells with CpG-ODN, with strong phosphorylation evident at 8 h. In contrast, IL-10 promoted STAT3 phosphorylation within 30 min and as expected, stimulation with IFNα promoted rapid STAT3 phosphorylation in HeLa cells within 5 min (Fig. 6C). Thus, the late activation of STAT3 in response to CpG-DNA may be a consequence of IL-10 production (mRNA levels peaked at 8 h, Fig. 4B).

Expression of a constitutively-active STAT3 mutant, STAT3C, in RAW cells potentiated luciferase expression driven by the S100A8 promoter (−178/465) without stimulation, and this significantly increased in response to CpG-ODN when compared to empty vector (pcDNA3) (1.43 and 1.85 fold respectively). In contrast, forced expression of dominant-negative STAT3β abolished the luciferase response to CpG-ODN, compared with the 2.2-fold induction by the corresponding empty vector pIRES-EGFP (Fig. 6D). To determine if STAT3 protein could directly bind to the putative −117 STAT3 binding site, ChIP assays were performed using primers flanking the region. STAT3 interacted with the defined promoter region in a time-dependent manner, with optimal interaction at 8 h that was maintained over 16 h (Fig. 6E). These results have identified a functional STAT3 binding site within the immediate promoter region of S100A8 in response to a TLR9 ligand.

## Discussion

This study shows strong S100A8 induction in macrophages by TLR9 ligands. S100A8 was directly induced by bacterial DNA or CpG-containing ODNs but not by human placental DNA; activation was negated by DNase treatment, or by an inhibitory CpG. This finding was replicated in primary murine peritoneal exudate macrophages and human M-CSF-differentiated macrophages. S100A9, which is co-expressed with S100A8 in high amounts in neutrophils, was not induced in murine macrophages, indicating distinct functions for S100A8 independent of the heterocomplex.

The S100A8/A9 complex is elevated in acute and chronic inflammatory lesions in humans, and systemic levels can indicate the extent of inflammation [41]. Mediators that dampen inflammation are generally induced later in macrophages [42] and in inflamed tissues, macrophages may comprise different activation states depending on the environmental stimuli. Macrophage deactivation by IL-10, TGF-β, glucocorticoid receptors, and cAMP via protein kinase A (PKA), generally promotes tissue repair and suppresses inflammation (reviewed in [43] and [2]). In macrophages, CpG-DNA-induced PGE_2_ and IL-10 inhibit IFN-γ [44] and IL-12 [35] responses respectively, thereby dampening Th1-responses. S100A8 induced by CpG-DNA required new protein synthesis, with kinetics much later than required for IL-10 or COX-2 mRNA expression and its induction was mediated in part by the secondary response gene products, IL-10 and PGE_2_ in an autocrine manner.

Although neither IL-10 nor PGE_2_ directly induced murine S100A8, they are also essential for its induction by LPS [23] and corticosteroids [22]; TGFβ and cAMP [19] also strongly synergize with LPS to increase expression. The critical role for IL-10 was confirmed earlier using macrophages from IL-10^−/−^ mice which failed to express S100A8 in response to LPS [23] or Poly I:C [20] although mechanisms are largely undefined. IL-10 has potent anti-inflammatory properties that suppress cytokine and chemokine production to limit host immune responses. Consistent with this, its suppressive effects are potentiated by its ability to induce other immunosuppressive products such as cytokine receptor antagonists [45]. Interestingly, like S100A8, induction of the IL-1 receptor antagonist, IL-1Ra by LPS in RAW 264.7 cells is enhanced by IL-10 although IL-10 had no direct effect [46].

PGE2 is also an important modulator of homeostasis and chronic inflammation (reviewed in [47]) with varying effects, depending on expression of particular PGE_2_ receptor (EP) subtypes, and through these, exerts pro- or anti-inflammatory functions. EP2 and EP4 receptors activate adenylate cyclase, increasing cAMP levels and PKA signaling [48]. In late/chronic inflammation, PGE2 has specialized anti-inflammatory properties by controlling potentially harmful over-activation of neutrophils, macrophages and lymphocytes, particularly Th1 cells producing IFNγ [49]. CpG DNA induces COX-2, promoting PGE_2_ production in RAW 264.7 macrophages [44], [50] and we found a requirement for this pathway in S100A8 induction. IL-10 or PGE_2_ enhanced CpG-induced S100A8 mRNA levels and shortened its maximal induction time by 12 h. Effects of IL-10 and COX-2 inhibitors on S100A8 and IL-10 were synergistic; S100A8 mRNA/protein expression was partially reduced by anti-IL-10, but when combined with a COX-2 inhibitor, mRNA levels were reduced by 96% and no secreted S100A8 was detected. PGE_2_ mediated effects were mainly through EP2 whereas IL-10 induction by PGE_2_ involved EP2 and EP4 (Fig. 4D). The COX2-PGE_2_-PKA pathway is also important for LPS, but not for poly I:C-induced S100A8 expression in macrophages [20] although mechanisms were unclear. RAW 264.7 cells express high levels of EP2 but this is much lower on peritoneal macrophages [51] and may explain the modest S100A8 induction observed with these (Fig. 1E). Furthermore, when p38, JNK, PI3K and JAK-dependent signaling required for S100A8 gene induction was inhibited, the response was only restored with IL-10 and PGE_2_ together, but not individually. PGE2 induces cAMP which promotes synergistic PKA- and p38-dependent IL-10 production in RAW 264.7 cells [52] and this pathway likely contributed to S100A8 induction in our study. CpG-DNA activates ERK, JNK, and p38 in RAW 264.7 cells to generate IL-10 [35]. Although the IL-10 and PGE_2_ pathways were both essential, they were not sufficient for CpG-DNA-mediated S100A8 induction. Neither substituted for CpG-DNA and did not directly induce S100A8 alone or combined [23], implicating additional pathways, possibly mediated by ERK1/2 activation (Fig. 5D). We are currently investigating this pathway. Notwithstanding, PGE_2_ activation of EP receptors initiates IL-10 gene induction and can also reprogram macrophages to an anti-inflammatory phenotype, indicating potentially important connections between PGE_2_, IL-10 (reviewed in [48]) and S100A8.

A small region (−178 to −34 bp from the transcription start site) was responsible for CpG-DNA-induced transcriptional activation of the S100A8 gene. NF-κB is implicated in IL-10 and COX-2 induction in murine macrophages [53], [54] but there is no NF-κB motif in this region and the NF-κB inhibitor (Bay 11–7082) did not inhibit the S100A8 luciferase response to CpG-DNA (data not shown), although the possibility that NF-κB may indirectly mediate S100A8 transcription by being required for generation of secondary mediators cannot be ruled out. CCAAT/enhancer binding protein β (C/EBPβ) is a cAMP-responsive transcription factor important for IL-10 induction in macrophages [55]. The essential promoter region of the murine S100A8 gene contains several C/EBPβ binding sites that are conserved in the human gene [14]. C/EBPβ is induced by CpG-DNA or PGE_2_ at early time, which involved in TLR3-mediated S100A8 induction in murine macrophages [20] and the human S100A8 promoter is activated by C/EBPβ [56]. IL-10 signaling also requires C/EBP proteins to promote its anti-inflammatory and immunomodulatory actions, including reduced IL-6 production in IL-10-treated human intestinal epithelial cells [57] and suppression of HIV-1 transcription in macrophages [58].

CpG-DNA-induced S100A8 did not totally overlap with S100A8 induction by LPS [20], or by Poly I:C [20]. Compared to LPS, the optimal time for S100A8 mRNA induction by CpG-DNA was earlier and less sustained. Moreover, the −94/465 reporter construct does not respond to LPS [23], but this generated a partial response to CpG-DNA (Fig. 6A). An NF-1 motif located −58 bp from the transcription start site is essential for synergy of LPS and glucocorticoids [22], but not for induction by LPS. The contribution of NF-1 to the CpG-DNA response requires further examination.

IL-10 regulates a large range of genes induced by LPS by dependent STAT3-dependent pathways [37] and the IL-10-JAK-STAT axis is critical for both acute and chronic inflammation [59]. Phospho-STAT3 was generated 8 h post-stimulation of RAW 267 cells with CpG, significantly later than phosphorylation induced by IL-10. We confirmed the importance of STAT3 recruitment using a luciferase expression system (Fig. 6) and demonstrated STAT3 binding to the defined promoter region, that was optimal at 8 h post-stimulation. Together, these results confirm a secondary response mediated by STAT3. We also located multiple C/EBP binding sites surrounding a unique STAT3 site in the essential promoter region of S100A8, similar to that identified in the neutrophil gelatinase-associated lipocalin promoter in macrophages that allowed its maximal induction by IL-10 [60]. The possibility that these transcription factors form complexes by binding different C/EBP binding sites in response to different stimuli, and potential interactions between STAT3 and C/EBP, or putative complex formation, warrant further investigation.

The combination of two stimuli, one a TLR ligand, to suppress macrophage cytokine induction is reported. A similar “strength of signal” model is proposed for IL-10 production itself, in T cells and macrophages [61], [62]. In this model, macrophage activation by a strong TLR agonist can induce pro-inflammatory cytokines, but is insufficient to produce high amounts of IL-10 and a second stimulus provides sufficient signaling strength for its production. In a manner similar to that reported here for S100A8, secondary modulators including adenosine, apoptotic cells, cAMP analogues, TGF-β, PGE2, and IL-10 can alter the balance to favour anti-inflammatory cytokine production, generated by so-called “regulatory macrophages” [62]. The main function of this macrophage type is to limit tissue damage. Despite the accepted role of S100A8 as a DAMP that propagates inflammation via interactions with TLR4 [63] or RAGE [64], the types of mediators involved in its induction in macrophages in response to TLR3, TLR4 and TLR9 ligands strongly implicate an immunomodulatory role. S100A8 down-regulates leukocyte adhesion and negatively regulates phagocyte transmigration [65], suppresses mast cell activation by scavenging ROS essential for signaling and inhibits acute murine asthma [29]. In addition, i.v. injection of S100A8/A9 into endotoxemic mice reduced neutrophil infiltration into organs and increased survival rates [66]. S100A8 is exquisitely sensitive to oxidation and may be critical in antioxidant defence in acute and chronic inflammation (reviewed in [67]). Indeed, we recently confirmed its ability to scavenge hypohalous acid oxidants in human asthma [27], and S-nitrosylated S100A8 inhibits leukocyte transmigration triggered by mast cell degranulation in the microcirculation [28]. Importantly, in cooperation with IL-10, S100A8 suppressed acute lung injury by suppressing mast-cell activation and leukocyte recruitment [68]. We propose that S100A8 contributes to anti-oxidant defence, particularly during the resolution phase of inflammation that is modulated by IL-10 and PGE2.

## Supporting Information

Figure S1 **Effects of selective JAK inhibitor Ruxolitinib on IL-10, Cox2 and S100A8 mRNA induction in RAW 264.7 cells.** RAW 264.7 cells were pretreated with DMSO (vehicle control) or Ruxolitinib (0.5 µg/ml) for 30 min then untreated or stimulated with CpG-ODN (3 µM) for 6 h (A and B), or 20 h (C). PGE_2_ (10 µM) and/or IL-10 (5 ng/ml) were added just before CpG-OND stimulation in (C). mRNA for IL-10 (A), COX-2 (B) or S100A8 (C) was quantitated using qRT-PCR. Results are means ± SD of 3 independent experiments. *P<0.05 or **P<0.01 of differences compared to CpG-ODN treatment along. Ns, not significant.
(TIF)

Table S1 **Ct values obtained by qRT-PCR assessment of murine S100A8, S100A9, and HPRT in macrophages and bone marrow cells.** RAW 264.7 cells were untreated (Med) or stimulated with CpG-ODN (CpG, 3 µM), LPS (20 ng/ml) or Poly I/C (pIC, 5 µM) for 20 h before harvesting. Bone marrow cells (BM) were obtained by flushing mouse femurs. mRNAs for S100A9, S100A8 or HPRT were quantitated using qRT-PCR. Ct values are presented in duplicate measurements of two separate samples. NAC, non-amplification control; UD, undetectable.
(DOCX)
