# Supplementary figures and images for: TLR9 Ligands Induce S100A8 in Macrophages via a STAT3-Dependent Pathway which Requires IL-10 and PGE2
Source: PLoS One. 2014 Aug 6;9(8):e103629. doi: 10.1371/journal.pone.0103629 (PMC4123874; doi:10.1371/journal.pone.0103629)

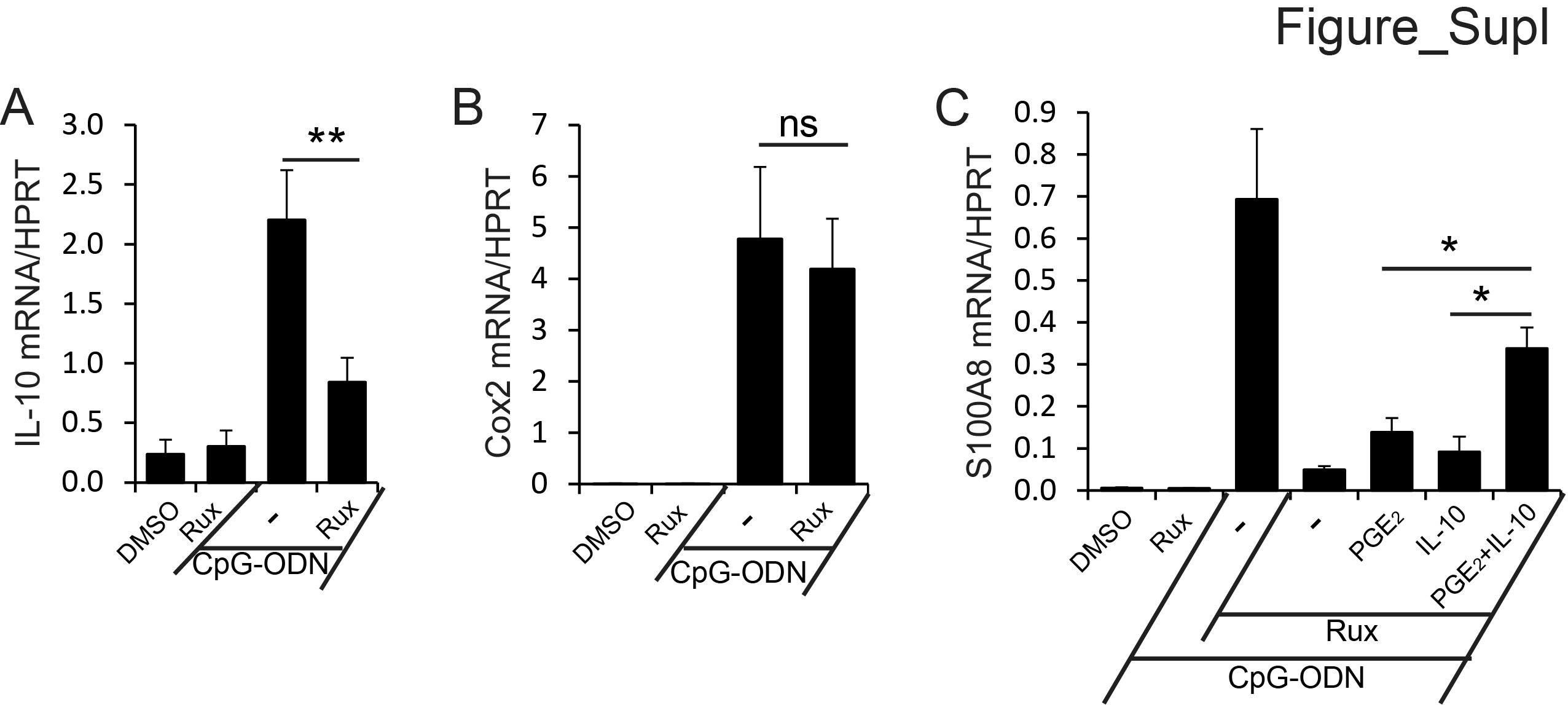

Supplement: Figure S1 — Effects of selective JAK inhibitor Ruxolitinib on IL-10, Cox2 and S100A8 mRNA induction in RAW 264.7 cells. RAW 264.7 cells were pretreated with DMSO (vehicle control) or Ruxolitinib (0.5 µg/ml) for 30 min then untreated or stimulated with CpG-ODN (3 µM) for 6 h (A and B), or 20 h (C). PGE2 (10 µM) and/or IL-10 (5 ng/ml) were added just before CpG-OND stimulation in (C). mRNA for IL-10 (A), COX-2 (B) or S100A8 (C) was quantitated using qRT-PCR. Results are means ± SD of 3 independent experiments. *P<0.05 or **P<0.01 of differences compared to CpG-ODN treatment along. Ns, not significant. (TIF) [file pone.0103629.s001.tif]
