# Supplementary material for: TLR9 Ligands Induce S100A8 in Macrophages via a STAT3-Dependent Pathway which Requires IL-10 and PGE2
Source: PLoS One. 2014 Aug 6;9(8):e103629. doi: 10.1371/journal.pone.0103629 (PMC4123874; doi:10.1371/journal.pone.0103629)
Supplement: Table S1 — Ct values obtained by qRT-PCR assessment of murine S100A8, S100A9, and HPRT in macrophages and bone marrow cells. RAW 264.7 cells were untreated (Med) or stimulated with CpG-ODN (CpG, 3 µM), LPS (20 ng/ml) or Poly I/C (pIC, 5 µM) for 20 h before harvesting. Bone marrow cells (BM) were obtained by flushing mouse femurs. mRNAs for S100A9, S100A8 or HPRT were quantitated using qRT-PCR. Ct values are presented in duplicate measurements of two separate samples. NAC, non-amplification control; UD, undetectable. (DOCX) [file pone.0103629.s002.docx]

|  | Med1 | Med2 | CpG1 | CpG2 | LPS1 | LPS2 | pIC1 | pIC2 | BM1 | BM2 | NAC |
| --- | --- | --- | --- | --- | --- | --- | --- | --- | --- | --- | --- |
| S100A9 | UD | UD | 36.99 | UD | UD | UD | UD | UD | 18.18 | 17.84 | UD |
|  | UD | UD | UD | UD | UD | UD | UD | UD | 17.23 | 17.82 | UD |
| S100A8 | 31.87 | 31.74 | 23.58 | 24.07 | 28.01 | 30.21 | 24.28 | 25.45 | 16.57 | 16.61 | UD |
|  | 31.65 | 32.35 | 23.61 | 24.19 | 28.24 | 30.88 | 24.26 | 25.07 | 16.47 | 16.21 | UD |
| HPRT | 23.86 | 24.61 | 23.33 | 23.75 | 24.74 | 26.62 | 25.07 | 25.84 | 24.61 | 24.95 | UD |
|  | 23.25 | 24.49 | 23.19 | 23.82 | 24.68 | 26.80 | 24.98 | 25.68 | 24.65 | 24.86 | UD |

Supplementary Table 1.
